# Supplementary material for: Computationally designed mRNA-launched protein nanoparticle vaccines
Source: bioRxiv. 2024 Jul 23:2024.07.22.604655. Preprint. [Version 1] doi: 10.1101/2024.07.22.604655 (PMC11291046; doi:10.1101/2024.07.22.604655)
Supplement: Supplement 4 [file NIHPP2024.07.22.604655v1-supplement-4.pdf]

bioRxiv preprint doi: https://doi.org/10.1101/2024.07.22.604655; this version posted July 23, 2024. The copyright holder for this preprint (which was not certified by peer review) is the author/funder, who has granted bioRxiv a license to display the preprint in perpetuity. It is made available under aCC-BY 4.0 International license.

Supplementary Table 1. Amino Acid Sequences of Proteins Used for Immunizations.

**>Rpk9-I3-01NS**<br>(MGILPSPGMPALLSLVSLLSVLLMGCVAETGT)RFPNITNLCPFGEVFNATRFASVYAWNRKRISNCVADFSVLYNSASFS<br>TFKCYGVSPTKLNDLCWTNIYADSFVIRGDEVRQIAPGQTGKIADYNYKLPDDFTGCVIAWNSNNLDSKVGGNYNYLYRL<br>FRKSNLKPFERDISTEIYQAGSTPCNGVEGFNCYFPLQSYGFQPTNGVGYQPYRVVVLSFELLHAPATVCGPKKSTGGS<br>GGSGSGGSGGSGSEKAAKAEEAARKMEELFKEHKIVAVLRANSVEEAKKKALAVFLGGVDLIEITFTVPDADTVIKELSFL<br>KEMGAIIGAGTVTSVEQAREAVESGAEFIVSPHLDEEISQFAKEEGVFYMPGVMTPTELVKAMKLGHTILKLFPGEVVGP<br>QFVEAMKGPFPNVKFVPTGGVNLDNVAEWFEAGVQAVGVGEALNEGTPVEVAEKAKAFVEKIEGATE
**>Rpk9-I53-50A**<br>(MGILPSPGMPALLSLVSLLSVLLMGCVAETGT)RFPNITNLCPFGEVFNATRFASVYAWNRKRISNCVADFSVLYNSASFS<br>TFKCYGVSPTKLNDLCWTNIYADSFVIRGDEVRQIAPGQTGKIADYNYKLPDDFTGCVIAWNSNNLDSKVGGNYNYLYRL<br>FRKSNLKPFERDISTEIYQAGSTPCNGVEGFNCYFPLQSYGFQPTNGVGYQPYRVVVLSFELLHAPATVCGPKKSTGGS<br>GGSGSGGSGGSGSEKAAKAEEAARKMEELFKKHKIVAVLRANSVEEAIEKAVAVFAGGVHLIEITFTVPDADTVIKALSVL<br>KEKGAIIGAGTVTSVEQARKAVESGAEFIVSPHLDEEISQFAKEKGVFYMPGVMTPTELVKAMKLGHTILKLFPGEVVGPQ<br>FVKAMKGPFPNVKFVPTGGVNLDNVAEWFKAGVLAVGVGSALVKGTPDEVREKAKAFVEKIRGATEGGSHHHHHHHH
**>S-2P-foldon**<br>(MGILPSPGMPALLSLVSLLSVLLMGCVAETGT)QCVNLTTRTQLPPAYTNSFTRGVYYPDKVFRSSVLHSTQDLFLPFFS<br>NVTWFHAIHVSGTNGTKRFDNPVLPFNDGVYFASTEKSNIIRGWIFGTTLDSKTQSLLIVNNATNVVIKVCEFQFCNDPFL<br>GVYYHKNNKSWMESEFRVYSSANNCTFEYVSQPFLMDLEGKQGNFKNLREFVFKNIDGYFKIYSKHTPINLVRDLPQGF<br>SALEPLVDLPIGINITRFQTLLALHRSYLTPGDSSSGWTAGAAAYYVGYLQPRTFLLKYNENGTITDAVDCALDPLSETKCT<br>LKSFTVEKGIYQTSNFRVQPTESIVRFPNITNLCPFGEVFNATRFASVYAWNRKRISNCVADYSVLYNSASFSTFKCYGVS<br>PTKLNDLCFTNVYADSFVIRGDEVRQIAPGQTGKIADYNYKLPDDFTGCVIAWNSNNLDSKVGGNYNYLYRLFRKSNLKP<br>FERDISTEIYQAGSTPCNGVEGFNCYFPLQSYGFQPTNGVGYQPYRVVVLSFELLHAPATVCGPKKSTNLVKNKCVNFN<br>FNGLTGTGVLTESNKKFLPFQQFGRDIADTTDAVRDPQTLEILDITPCSFGGVSVITPGTNTSNQVAVLYQDVNCTEVPVAI<br>HADQLTPTWRVYSTGSNVFQTRAGCLIGAEHVNNSYECDIPIGAGICASYQTQTNSPSGAGSVASQSIIAYTMSLGAENS<br>VAYSNNSIAIPTNFTISVTTEILPVSMTKTSVDCTMYICGDSTECSNLLLQYGSFCTQLNRALTGIAVEQDKNTQEVFAQVK<br>QIYKTPPIKDFGGFNFSQILPDPSKPSKRSFIEDLLFNKVTLADAGFIKQYGDCLGDIAARDLICAQKFNGLTVLPPLLTDEM<br>IAQYTSALLAGTITSGWTFGAGAALQIPFAMQMAYRFNGIGVTQNVLYENQKLIANQFNSAIGKIQDSLSSTASALGKLQD<br>VVNQNAQALNTLVKQLSSNFGAISSVLNDILSRLDPPEAEVQIDRLITGRLQSLQTYVTQQLIRAAEIRASANLAATKMSEC<br>VLGQSKRVDFCGKGYHLMSFPQSAPHGVVFLHVTYVPAQEKNFTTAPAICHDGKAHFPREGVFVSNGTHWFVTQRNFY<br>EPQIITTDNTFVSGNCDVVIGIVNNTVYDPLQPELDSFKEELDKYFKNHTSPDVDLGDISGINASVVNIQKEIDRLNEVAKN<br>LNESLIDLQELGKYEQYIKGSGRENLYFQGGGGSGYIPEAPRDGQAYVRKDGEWVLLSTFLGHHHHHHHH
**>Rpk9-I53-50 (A component : B component)**<br>(MGILPSPGMPALLSLVSLLSVLLMGCVAETGT)RFPNITNLCPFGEVFNATRFASVYAWNRKRISNCVADFSVLYNSASFS<br>TFKCYGVSPTKLNDLCWTNIYADSFVIRGDEVRQIAPGQTGKIADYNYKLPDDFTGCVIAWNSNNLDSKVGGNYNYLYRL<br>FRKSNLKPFERDISTEIYQAGSTPCNGVEGFNCYFPLQSYGFQPTNGVGYQPYRVVVLSFELLHAPATVCGPKKSTGGS<br>GGSGSGGSGGSGSEKAAKAEEAARKMEELFKKHKIVAVLRANSVEEAIEKAVAVFAGGVHLIEITFTVPDADTVIKALSVL<br>KEKGAIIGAGTVTSVEQARKAVESGAEFIVSPHLDEEISQFAKEKGVFYMPGVMTPTELVKAMKLGHTILKLFPGEVVGPQ<br>FVKAMKGPFPNVKFVPTGGVNLDNVAEWFKAGVLAVGVGSALVKGTPDEVREKAKAFVEKIRGATEGGSHHHHHHHH :<br>MNQHSHKDHETVRIAVVRARWHAEIVDACVSAFEAAMRDIGGDRFAVDVFDVPGAYEIPLHARTLAETGRYGAVLGTAF<br>VVNGGIYRHEFVASAVINGMMNVQLNTGVPVLSAVLTPHNYDKSKAHTLLFLALFAVKGMEAARACVEILAAREKIAA

Signal peptides are indicated in parentheses.

bioRxiv preprint doi: https://doi.org/10.1101/2024.07.22.604655; this version posted July 23, 2024. The copyright holder for this preprint (which was not certified by peer review) is the author/funder, who has granted bioRxiv a license to display the preprint in perpetuity. It is made available under aCC-BY 4.0 International license.

Supplementary Table 2. ORF Nucleic Acid Sequences of mRNA Constructs Used for Immunizations.

>Rpk9-I3-01NS

AΨGGGCAΨCCΨGCCAAGCCCΨGGAAΨGCCΨGCCCΨGCΨGAGCCΨGGΨGΨCCCΨGCΨGΨCΨGΨGCΨGCΨG
AΨGGGAΨGCGΨGGCA)GAGACCGGCACAAGGΨΨCCCΨAACAΨCACCAAΨCΨGΨGCCCAΨΨCGGCGAGGΨGΨ
ΨΨAACGCCACACGCΨΨΨGCCΨCCGΨGΨAΨGCCΨGGAACCGGAAGAGAAΨCΨCΨAAΨΨGCGΨGGCCGACΨΨ
CAGCGΨGCΨGΨACAAΨAGCGCCΨCCΨΨCΨCΨACCΨΨΨAAGΨGCΨAΨGGCGΨGAGCCCCACCAAGCΨGAAC
GAΨCΨGΨGCΨGGACAAACAΨΨΨACGCCGACΨCCΨΨΨGΨGAΨCCGGGGCGAΨGAAGΨGAGACAGAΨCGCAC
CAGGACAGACCGGAAAGAΨCGCAGACΨACAACΨAΨAAGCΨGCCΨGACGAΨΨΨCACAGGCΨGCGΨGAΨCGCC
ΨGGAAΨAGCAACAAΨCΨGGAΨΨCCAAAGΨGGGCGGCAACΨACAAΨΨAΨCΨGΨACAGGCΨGΨΨCCGCAAGΨ
CCAACCΨGAAGCCAΨΨΨGAGCGGGACAΨCΨCCACCGAGAΨCΨACCAGGCCGGCΨCΨACACCCΨGCAACGGC
GΨGGAGGGCΨΨCAAΨΨGΨΨAΨΨΨΨCCCCΨGCAGΨCΨΨACGGCΨΨCCAGCCΨACAAAΨGGCGΨGGGCΨAΨ
CAGCCAΨACCGGGΨGGΨGGΨGCΨGΨCΨΨΨΨGAGCΨGCΨGCACGCACCAGCAACCGΨGΨGCGGCCCCAAGA
AGAGCACAGGAGGCΨCΨGGAGGCAGCGGCΨCCGGAGGCΨCΨGGAGGCAGCGGCΨCCGAGAAGGCCGCCAAG
GCCGAGGAGGCCGCCAGGAAGAΨGGAGGAGCΨCΨΨΨAAGGAGCACAAAAΨCGΨGGCGGΨGCΨCAGGGCAAA
ΨAGCGΨAGAAGAGGCCAAAAAGAAGGCACΨCGCCGΨCΨΨΨCΨCGGAGGCGΨGGACCΨGAΨAGAGAΨCACAΨ
ΨCACGGΨGCCAGAΨGCΨGAΨACGGΨCAΨΨAAGGAGCΨGAGΨΨΨΨCΨCAAAGAGAΨGGGCGCCAΨCAΨAGG
CGCCGGCACCGΨGACAΨCCGΨGGAGCAGGCCAGAGAAGCCGΨCGAGΨCCGGAGCCGAAΨΨCAΨAGΨGΨCCC
CΨCAΨCΨCGAΨGAGGAGAΨCAGΨCAGΨΨΨGCCAAGGAGGAGGGAGΨGΨΨΨΨAΨAΨGCCCGGAGΨGAΨGAC
GCCGACGGAGCΨAGΨGAAGGCCAΨGAAACΨGGGCCACACCAΨCCΨCAAGCΨGΨΨΨCCΨGGAGAAGΨGGΨΨ
GGΨCCCCAAΨΨΨGΨCGAAGCCAΨGAAGGGACCAΨΨΨCCAAACGΨGAAAΨΨCGΨGCCGACCGGAGGAGΨΨAA
CΨΨAGACAACGΨΨGCCGAGΨGGΨΨΨGAGGCAGGCGΨΨCAGGCGGΨCGGGGΨCGGAGAGGCCCΨCAACGAA
GGAACACCAGΨGGAAGΨCGCCGAGAAGGCCAAAGCAΨΨΨGΨGGAGAAAAΨCGAGGGAGCCACΨGAAΨGAΨG
A

>Rpk9-I53-50A

AΨGGGCAΨCCΨGCCAAGCCCΨGGAAΨGCCΨGCCCΨGCΨGAGCCΨGGΨGΨCCCΨGCΨGΨCΨGΨGCΨGCΨG
AΨGGGAΨGCGΨGGCA)GAGACCGGCACAAGGΨΨCCCΨAACAΨCACCAAΨCΨGΨGCCCAΨΨCGGCGAGGΨGΨ
ΨΨAACGCCACACGCΨΨΨGCCΨCCGΨGΨAΨGCCΨGGAACCGGAAGAGAAΨCΨCΨAAΨΨGCGΨGGCCGACΨΨ
CAGCGΨGCΨGΨACAAΨAGCGCCΨCCΨΨCΨCΨACCΨΨΨAAGΨGCΨAΨGGCGΨGAGCCCCACCAAGCΨGAAC
GAΨCΨGΨGCΨGGACAAACAΨΨΨACGCCGACΨCCΨΨΨGΨGAΨCCGGGGCGAΨGAAGΨGAGACAGAΨCGCAC
CAGGACAGACCGGAAAGAΨCGCAGACΨACAACΨAΨAAGCΨGCCΨGACGAΨΨΨCACAGGCΨGCGΨGAΨCGCC
ΨGGAAΨAGCAACAAΨCΨGGAΨΨCCAAAGΨGGGCGGCAACΨACAAΨΨAΨCΨGΨACAGGCΨGΨΨCCGCAAGΨ
CCAACCΨGAAGCCAΨΨΨGAGCGGGACAΨCΨCCACCGAGAΨCΨACCAGGCCGGCΨCΨACACCCΨGCAACGGC
GΨGGAGGGCΨΨCAAΨΨGΨΨAΨΨΨΨCCCCΨGCAGΨCΨΨACGGCΨΨCCAGCCΨACAAAΨGGCGΨGGGCΨAΨ
CAGCCAΨACCGGGΨGGΨGGΨGCΨGΨCΨΨΨΨGAGCΨGCΨGCACGCACCAGCAACCGΨGΨGCGGCCCCAAGA
AGAGCACAGGAGGCΨCΨGGAGGCAGCGGCΨCCGGAGGCΨCΨGGAGGCAGCGGCΨCCGAGAAGGCCGCCAAG
GCCGAGGAGGCCGCCAGGAAGAΨGGAGGAGCΨGΨΨCAAGAAGCACAAGAΨCGΨGGCCGΨGCΨGAGGGCCAA
CAGCGΨGGAGGAGGCCAΨCGAGAAGGCAGΨGGCCGΨGΨΨCGCAGGAGGAGΨGCACCΨGAΨCGAGAΨCACC
ΨΨΨACAGΨGCCAGACGCCGAΨACCGΨGAΨCAAGGCCCΨGΨCCGΨGCΨGAAGGAGAAGGGAGCAAΨCAΨCG
GAGCAGGCACCGΨGACAΨCΨGΨGGAGCAGGCAAGGAAGGCAGΨGGAGΨCΨGGAGCCGAGΨΨΨAΨCGΨGAG
CCCCCACCΨGGACGAGGAGAΨCΨCCCAGΨΨCGCCAAGGAGAAGGGCGΨGΨΨΨΨACAΨGCCAGGCGΨGAΨG
ACCCCCACAGAGCΨGGΨGAAGGCCAΨGAAGCΨGGGCCACACCAΨCCΨGAAGCΨGΨΨCCCΨGGCGAGGΨGG
ΨGGGACCCCAGΨΨΨGΨGAAGGCCAΨGAAAGGCCCCΨΨCCCΨAAΨGΨGAAGΨΨΨGΨGCCCACAGGCGGCGΨ
GAACCΨGGACAAΨGΨGGCAGAGΨGGΨΨCAAGGCAGGCGΨGCΨGGCCGΨGGGAGΨGGGCAGCGCCCΨGGΨ
GAAGGGCACCCCΨGAΨGAGGΨGCGGGAGAAGGCCAAGGCCΨΨΨGΨGGAGAAGAΨCAGAGGCGCCACAGAGΨ
GAΨGA

>Membrane-anchored S-2P

bioRxiv preprint doi: https://doi.org/10.1101/2024.07.22.604655; this version posted July 23, 2024. The copyright holder for this preprint (which was not certified by peer review) is the author/funder, who has granted bioRxiv a license to display the preprint in perpetuity. It is made available under aCC-BY 4.0 International license.

AΨGΨΨCGΨGΨΨCCΨGGΨGCΨGCΨGCCΨCΨGGΨGΨCCAGCCAGΨGΨGΨGAACCΨGACCACCAGAACACAG
CΨGCCΨCCAGCCΨACACCAACAGCΨΨΨACCAGAGGCGΨGΨACΨACCCCGACAAGGΨGΨΨCAGAΨCCAGCGΨ
GCΨGCACΨCΨACCCAGGACCΨGΨΨCCΨGCCΨΨΨCΨΨCAGCAACGΨGACCΨGGΨΨCCACGCCAΨCCACGΨG
ΨCCGGCACCAAΨGGCACCAAGAGAΨΨCGACAACCCCGΨGCΨGCCCΨΨCAACGACGGGGΨGΨACΨΨΨGCCAG
CACCGAGAAGΨCCAACAΨCAΨCAGAGGCΨGGAΨCΨΨCGGCACCACACΨGGACAGCAAGACCCAGAGCCΨGCΨ
GAΨCGΨGAACAACGCCACCAACGΨGGΨCAΨCAAAGΨGΨGCGAGΨΨCCAGΨΨCΨGCAACGACCCCΨΨCCΨG
GGCGΨCΨACΨACCACAAGAACAACAAGAGCΨGGAΨGGAAAGCGAGΨΨCCGGGΨGΨACAGCAGCGCCAACAAC
ΨGCACCΨΨCGAGΨACGΨGΨCCCAGCCΨΨΨCCΨGAΨGGACCΨGGAAGGCAAGCAGGGCAACΨΨCAAGAACC
ΨGCGCGAGΨΨCGΨGΨΨΨAAGAACAΨCGACGGCΨACΨΨCAAGAΨCΨACAGCAAGCACACCCCΨAΨCAACCΨC
GΨGCGGGAΨCΨGCCΨCAGGGCΨΨCΨCΨGCΨCΨGGAACCCCΨGGΨGGAΨCΨGCCCAΨCGGCAΨCAACAΨCA
CCCGGΨΨΨCAGACACΨGCΨGGCCCΨGCACAGAAGCΨACCΨGACACCΨGGCGAΨAGCAGCAGCGGAΨGGACA
GCΨGGΨGCCGCCGCΨΨACΨAΨGΨGGGCΨACCΨGCAGCCΨAGAACCΨΨCCΨGCΨGAAGΨACAACGAGAACG
GCACCAΨCACCGACGCCGΨGGAΨΨGΨGCΨCΨGGAΨCCΨCΨGAGCGAGACAAAGΨGCACCCΨGAAGΨCCΨΨ
CACCGΨGGAAAAGGGCAΨCΨACCAGACCAGCAACΨΨCCGGGΨGCAGCCCACCGAAΨCCAΨCGΨGCGGΨΨCC
CCAAΨAΨCACCAAΨCΨGΨGCCCCΨΨCGGCGAGGΨGΨΨCAAΨGCCACCAGAΨΨCGCCΨCΨGΨGΨACGCCΨG
GAACCGGAAGCGGAΨCAGCAAΨΨGCGΨGGCCGACΨACΨCCGΨGCΨGΨACAACΨCCGCCAGCΨΨCAGCACCΨ
ΨCAAGΨGCΨACGGCGΨGΨCCCCΨACCAAGCΨGAACGACCΨGΨGCΨΨCACAAACGΨGΨACGCCGACAGCΨΨ
CGΨGAΨCCGGGGAGAΨGAAGΨGCGGCAGAΨΨGCCCCΨGGACAGACAGGCAAGAΨCGCCGACΨACAACΨACA
AGCΨGCCCGACGACΨΨCACCGGCΨGΨGΨGAΨΨGCCΨGGAACAGCAACAACCΨGGACΨCCAAAGΨCGGCGGC
AACΨACAAΨΨACCΨGΨACCGGCΨGΨΨCCGGAAGΨCCAAΨCΨGAAGCCCΨΨCGAGCGGGACAΨCΨCCACCGA
GAΨCΨAΨCAGGCCGGCAGCACCCCΨΨGΨAACGGCGΨGGAAGGCΨΨCAACΨGCΨACΨΨCCCACΨGCAGΨCC
ΨACGGCΨΨΨCAGCCCACAAAΨGGCGΨGGGCΨAΨCAGCCCΨACAGAGΨGGΨGGΨGCΨGAGCΨΨCGAACΨGC
ΨGCAΨGCCCCΨGCCACAGΨGΨGCGGCCCΨAAGAAAAGCACCAAΨCΨCGΨGAAGAACAAAΨGCGΨGAACΨΨC
AACΨΨCAACGGCCΨGACCGGCACCGGCGΨGCΨGACAGAGAGCAACAAGAAGΨΨCCΨGCCAΨΨCCAGCAGΨΨ
ΨGGCCGGGAΨAΨCGCCGAΨACCACAGACGCCGΨΨAGAGAΨCCCCAGACACΨGGAAAΨCCΨGGACAΨCACCC
CΨΨGCAGCΨΨCGGCGGAGΨGΨCΨGΨGAΨCACCCCΨGGCACCAACACCAGCAAΨCAGGΨGGCAGΨGCΨGΨA
CCAGGACGΨGAACΨGΨACCGAAGΨGCCCGΨGGCCAΨΨCACGCCGAΨCAGCΨGACACCΨACAΨGGCGGGΨG
ΨACΨCCACCGGCAGCAAΨGΨGΨΨΨCAGACCAGAGCCGGCΨGΨCΨGAΨCGGAGCCGAGCACGΨGAACAAΨAG
CΨACGAGΨGCGACAΨCCCCAΨCGGCGCΨGGAAΨCΨGCGCCAGCΨACCAGACACAGACAAACAGCCCΨCGGAG
AGCCAGAAGCGΨGGCCAGCCAGAGCAΨCAΨΨGCCΨACACAAΨGΨCΨCΨGGGCGCCGAGAACAGCGΨGGCCΨ
ACΨCCAACAACΨCΨAΨCGCΨAΨCCCCACCAACΨΨCACCAΨCAGCGΨGACCACAGAGAΨCCΨGCCΨGΨGΨCC
AΨGACCAAGACCAGCGΨGGACΨGCACCAΨGΨACAΨCΨGCGGCGAΨΨCCACCGAGΨGCΨCCAACCΨGCΨGCΨ
GCAGΨACGGCAGCΨΨCΨGCACCCAGCΨGAAΨAGAGCCCΨGACAGGGAΨCGCCGΨGGAACAGGACAAGAACA
CCCAAGAGGΨGΨΨCGCCCAAGΨGAAGCAGAΨCΨACAAGACCCCΨCCΨAΨCAAGGACΨΨCGGCGGCΨΨCAAΨ
ΨΨCAGCCAGAΨΨCΨGCCCGAΨCCΨAGCAAGCCCAGCAAGCGGAGCΨΨCAΨCGAGGACCΨGCΨGΨΨCAACAA
AGΨGACACΨGGCCGACGCCGGCΨΨCAΨCAAGCAGΨAΨGGCGAΨΨGΨCΨGGGCGACAΨΨGCCGCCAGGGAΨ
CΨGAΨΨΨGCGCCCAGAAGΨΨΨAACGGACΨGACAGΨGCΨGCCΨCCΨCΨGCΨGACCGAΨGAGAΨGAΨCGCCC
AGΨACACAΨCΨGCCCΨGCΨGGCCGGCACAAΨCACAAGCGGCΨGGACAΨΨΨGGAGCAGGCGCCGCΨCΨGCA
GAΨCCCCΨΨΨGCΨAΨGCAGAΨGGCCΨACCGGΨΨCAACGGCAΨCGGAGΨGACCCAGAAΨGΨGCΨGΨACGAG
AACCAGAAGCΨGAΨCGCCAACCAGΨΨCAACAGCGCCAΨCGGCAAGAΨCCAGGACAGCCΨGAGCAGCACAGCA
AGCGCCCΨGGGAAAGCΨGCAGGACGΨGGΨCAACCAGAAΨGCCCAGGCACΨGAACACCCΨGGΨCAAGCAGCΨ
GΨCCΨCCAACΨΨCGGCGCCAΨCAGCΨCΨGΨGCΨGAACGAΨAΨCCΨGAGCAGACΨGGACCCΨCCΨGAGGCC
GAGGΨGCAGAΨCGACAGACΨGAΨCACAGGCAGACΨGCAGAGCCΨCCAGACAΨACGΨGACCCAGCAGCΨGAΨ
CAGAGCCGCCGAGAΨΨAGAGCCΨCΨGCCAAΨCΨGGCCGCCACCAAGAΨGΨCΨGAGΨGΨGΨGCΨGGGCCAG
AGCAAGAGAGΨGGACΨΨΨΨGCGGCAAGGGCΨACCACCΨGAΨGAGCΨΨCCCΨCAGΨCΨGCCCCΨCACGGCG
ΨGGΨGΨΨΨCΨGCACGΨGACAΨAΨGΨGCCCGCΨCAAGAGAAGAAΨΨΨCACCACCGCΨCCAGCCAΨCΨGCCA
CGACGGCAAAGCCCACΨΨΨCCΨAGAGAAGGCGΨGΨΨCGΨGΨCCAACGGCACCCAΨΨGGΨΨCGΨGACACAG
CGGAACΨΨCΨACGAGCCCCAGAΨCAΨCACCACCGACAACACCΨΨCGΨGΨCΨGGCAACΨGCGACGΨCGΨGAΨ

bioRxiv preprint doi: https://doi.org/10.1101/2024.07.22.604655; this version posted July 23, 2024. The copyright holder for this preprint (which was not certified by peer review) is the author/funder, who has granted bioRxiv a license to display the preprint in perpetuity. It is made available under aCC-BY 4.0 International license.

CGGCAΨΨGΨGAACAAΨACCGΨGΨACGACCCΨCΨGCAGCCCGAGCΨGGACAGCΨΨCAAAGAGGAACΨGGACA<br>AGΨACΨΨΨAAGAACCACACAAGCCCCGACGΨGGACCΨGGGCGAΨAΨCAGCGGAAΨCAAΨGCCAGCGΨCGΨG<br>AACAΨCCAGAAAGAGAΨCGACCGGCΨGAACGAGGΨGGCCAAGAAΨCΨGAACGAGAGCCΨGAΨCGACCΨGCAA<br>GAACΨGGGGAAGΨACGAGCAGΨACAΨCAAGΨGGCCCΨGGΨACAΨCΨGGCΨGGGCΨΨΨAΨCGCCGGACΨGA<br>ΨΨGCCAΨCGΨGAΨGGΨCACAAΨCAΨGCΨGΨGΨΨGCAΨGACCAGCΨGCΨGΨAGCΨGCCΨGAAGGGCΨGΨΨ<br>GΨAGCΨGΨGGCAGCΨGCΨGCAAGΨΨCGACGAGGACGAΨΨCΨGAGCCCGΨGCΨGAAGGGCGΨGAAACΨGCA<br>CΨACACAΨGAΨGA
**>Luciferase**<br>AΨGGAAGAΨGCCAAAAACAΨΨAAGAAGGGCCCAGCGCCAΨΨCΨACCCACΨCGAAGACGGGACCGCCGGCGAG<br>CAGCΨGCACAAAGCCAΨGAAGCGCΨACGCCCΨGGΨGCCCGGCACCAΨCGCCΨΨΨACCGACGCACAΨAΨCGA<br>GGΨGGACAΨΨACCΨACGCCGAGΨACΨΨCGAGAΨGAGCGΨΨCGGCΨGGCAGAAGCΨAΨGAAGCGCΨAΨGGG<br>CΨGAAΨACAAACCAΨCGGAΨCGΨGGΨGΨGCAGCGAGAAΨAGCΨΨGCAGΨΨCΨΨCAΨGCCCGΨGΨΨGGGΨ<br>GCCCΨGΨΨCAΨCGGΨGΨGGCΨGΨGGCCCCAGCΨAACGACAΨCΨACAACGAGCGCGAGCΨGCΨGAACAGCA<br>ΨGGGCAΨCAGCCAGCCCACCGΨCGΨAΨΨCGΨGAGCAAGAAAGGGCΨGCAAAAGAΨCCΨCAACGΨGCAAAAG<br>AAGCΨACCGAΨCAΨACAAAAGAΨCAΨCAΨCAΨGGAΨAGCAAGACCGACΨACCAGGGCΨΨCCAAAGCAΨGΨAC<br>ACCΨΨCGΨGACΨΨCCCAΨΨΨGCCACCCGGCΨΨCAACGAGΨACGACΨΨCGΨGCCCGAGAGCΨΨCGACCGGG<br>ACAAAACCAΨCGCCCΨGAΨCAΨGAACAGΨAGΨGGCAGΨACCGGAΨΨGCCCAAGGGCGΨAGCCCΨACCGCAC<br>CGCACCGCΨΨGΨGΨCCGAΨΨCAGΨCAΨGCCCGCGACCCCAΨCΨΨCGGCAACCAGAΨCAΨCCCCGACACCGC<br>ΨAΨCCΨCAGCGΨGGΨGCCAΨΨΨCACCACGGCΨΨCGGCAΨGΨΨCACCACGCΨGGGCΨACΨΨGAΨCΨGCGG<br>CΨΨΨCGGGΨCGΨGCΨCAΨGΨACCGCΨΨCGAGGAGGAGCΨAΨΨCΨΨGCGCAGCΨΨGCAAGACΨAΨAAGAΨ<br>ΨCAAΨCΨGCCCΨGCΨGGΨGCCCACACΨAΨΨΨAGCΨΨCΨΨCGCΨAAGAGCACΨCΨCAΨCGACAAGΨACGAC<br>CΨAAGCAACΨΨGCACGAGAΨCGCCAGCGGCGGGGCGCCGCΨCAGCAAGGAGGΨAGGΨGAGGCCGΨGGCCAA<br>ACGCΨΨCCACCΨACCAGGCAΨCCGACAGGGCΨACGGCCΨGACAGAAACAACCAGCGCCAΨΨCΨGAΨCACCCC<br>CGAAGGGGACGACAAGCCΨGGCGCAGΨAGGCAAGGΨGGΨGCCCΨΨCΨΨCGAGGCΨAAGGΨGGΨGGACΨΨG<br>GACACCGGΨAAGACACΨGGGΨGΨGAACCAGCGCGGCGAGCΨGΨGCGΨCCGΨGGCCCCAΨGAΨCAΨGAGCG<br>GCΨACGΨΨAACAACCCCGAGGCΨACAAACGCΨCΨCAΨCGACAAGGACGGCΨGGCΨGCACAGCGGCGACAΨC<br>GCCΨACΨGGGACGAGGACGAGCACΨΨCΨΨCAΨCGΨGGACCGGCΨGAAGΨCCCΨGAΨCAAAΨACAAGGGCΨ<br>ACCAGGΨAGCCCCAGCCGAACΨGGAGAGCAΨCCΨGCΨGCAACACCCCAACAΨCΨΨCGACGCCGGGGΨCGCC<br>GGCCΨGCCCGACGACGAΨGCCGGCGAGCΨGCCCGCCGCAGΨCGΨCGΨGCΨGGAACACGGΨAAAACCAΨGAC<br>CGAGAAGGAGAΨCGΨGGACΨAΨGΨGGCCAGCCAGGΨΨACAACCGCCAAGAAGCΨGCGCGGΨGGΨGΨΨGΨG<br>ΨΨCGΨGGACGAGGΨGCCΨAAAGGACΨGACCGGCAAGΨΨGGACGCCCGCAAGAΨCCGCGAGAΨΨCΨCAΨΨA<br>AGGCCAAGAAGGGCGGCAAGAΨCGCCGΨG

Ψ, N1-methylpseudouridine-5'-triphosphate
